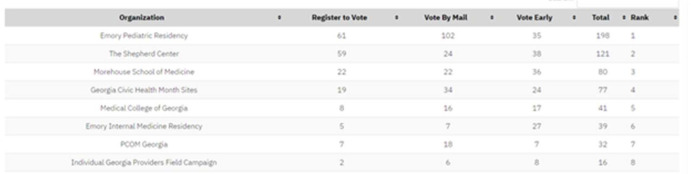# Vot-ER

**DOI:** 10.5195/jmla.2021.1197

**Published:** 2021-04-01

**Authors:** Jess Callaway

**Affiliations:** 1 jessica.callaway@shepherd.org, Library Director for the Noble Learning Research Center, Shepherd Center, Atlanta, GA

## INTRODUCTION

Vot-ER is a nonpartisan “team of physicians, designers, and behavioral scientists committed to helping make it easier for patients to participate in the democratic process” [1]. Vot-ER's core beliefs focus on healthcare, community, and democracy and are as follows:

- “Social Determinants of Health: We believe that empowered voices in communities leads to positive health outcomes.
- Voting Gives Us Voice: We believe that by voting for our health, we can preserve what works or demand change for the things that don’t.
- More Voices Build Better Systems: We believe our healthcare system is stronger when we, as providers and patients, all show up” [2].

The COVID-19 pandemic and the 2020 presidential election presented unique civic barriers for many people. This review will focus on the barriers experienced by two groups of people: healthcare providers and patients, and how Vot-ER is a good solution for this problem.

## MISSION

Vot-ER 's goal is to “provide patients the opportunity to register to vote, because much of our healthcare system and healthcare experiences are determined by the policies our elected officials implement” [1]. Additionally, Vot-ER recognizes that “hospitals and community health clinics, like schools, DMVs, and libraries, are central touchpoints in communities, where citizens should consider their civic health as well as their physical and mental health” [1].

## TOOLS

Vot-ER provides many useful free resources to assist patients with voting while in a healthcare setting, utilizing QR codes and visual cues.

### Healthy Democracy Kit

**Figure 1:**
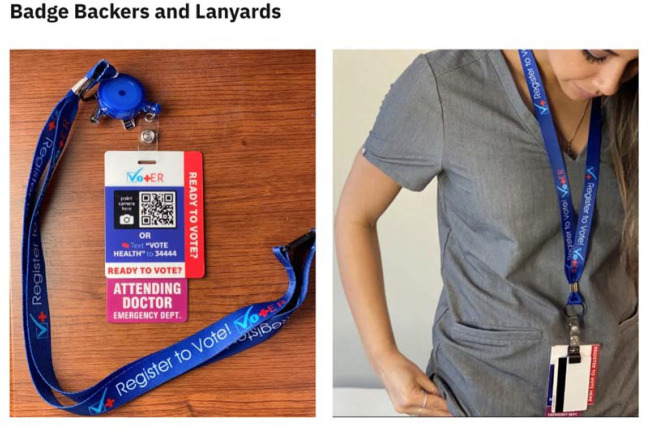
Healthy Democracy Kit

The Healthy Democracy Kit contains a lanyard and badge backer with a QR code for scanning that opens up to a website which assists with either registering to vote or requesting a ballot by mail (Figure 1). The QR code is easily scanned using a cellular phone. These kits can be ordered free of charge and can be customized to include institutional branding.

### Site-Based Setup

“Our Touchless offering includes a set of posters designed to excite patients about registering to vote, a sign, stickers, and handouts. The resources are in English and Spanish” [3].

### Telehealth Kits

“Vot-ER has been adapted to telehealth platforms using links and text short codes to quickly connect people to state based voter registration” [4].

### Scripting

Vot-ER makes it easy to start conversations with patients and other care providers by offering a library of scripting that can be used in most situations that may arise [5].

## IMPACT

Depending on the type of Healthy Democracy Kit you order, Vot-ER offers impact tracking so you can monitor your work and compare your registration rates to other systems in your local area by utilizing the state leadership board. This feature is offered for kits that have institutional branding. At this reviewer's institution, this was a valuable resource and has been included in the library yearly impact score.

## CONCLUSION

In a year like no other, Vot-ER made it easy to provide a safe way for patients to vote. This is a reliable and easy to use resource that can easily be incorporated into hospital library outreach programs. The Vot-ER team is very responsive and happy to help with customizations that will better serve all populations, including clinical staff, medical students, and patients.

For librarians, this offers a chance to provide crucial civic outreach at no cost to patients and clinical staff alike, as literature shows that medical professionals vote at a lower rate than the general population [6, 7]. At this reviewer's institution, as displayed in Table 1, a system-wide email with Vot-ER's information and links to the easy-to-use voter registration platform resulted in more than 100 staff securing their ballot for a runoff election in a single day. Ultimately, this is a resource that is appropriate for librarians in a wide variety of healthcare settings.

**Table 1:** Metrics for reporting participation